# Supplementary material for: Prior knowledge informs graph neural networks to improve phenotype prediction from proteomics
Source: medRxiv. 2025 Nov 25:2025.11.23.25340814. Preprint. [Version 1] doi: 10.1101/2025.11.23.25340814 (PMC12676398; doi:10.1101/2025.11.23.25340814)
Supplement: 1 [file NIHPP2025.11.23.25340814V1-supplement-1.pdf]

medRxiv preprint doi: https://doi.org/10.1101/2025.11.23.25340814; this version posted November 25, 2025. The copyright holder for this preprint (which was not certified by peer review) is the author/funder, who has granted medRxiv a license to display the preprint in perpetuity.

It is made available under a CC-BY 4.0 International license .

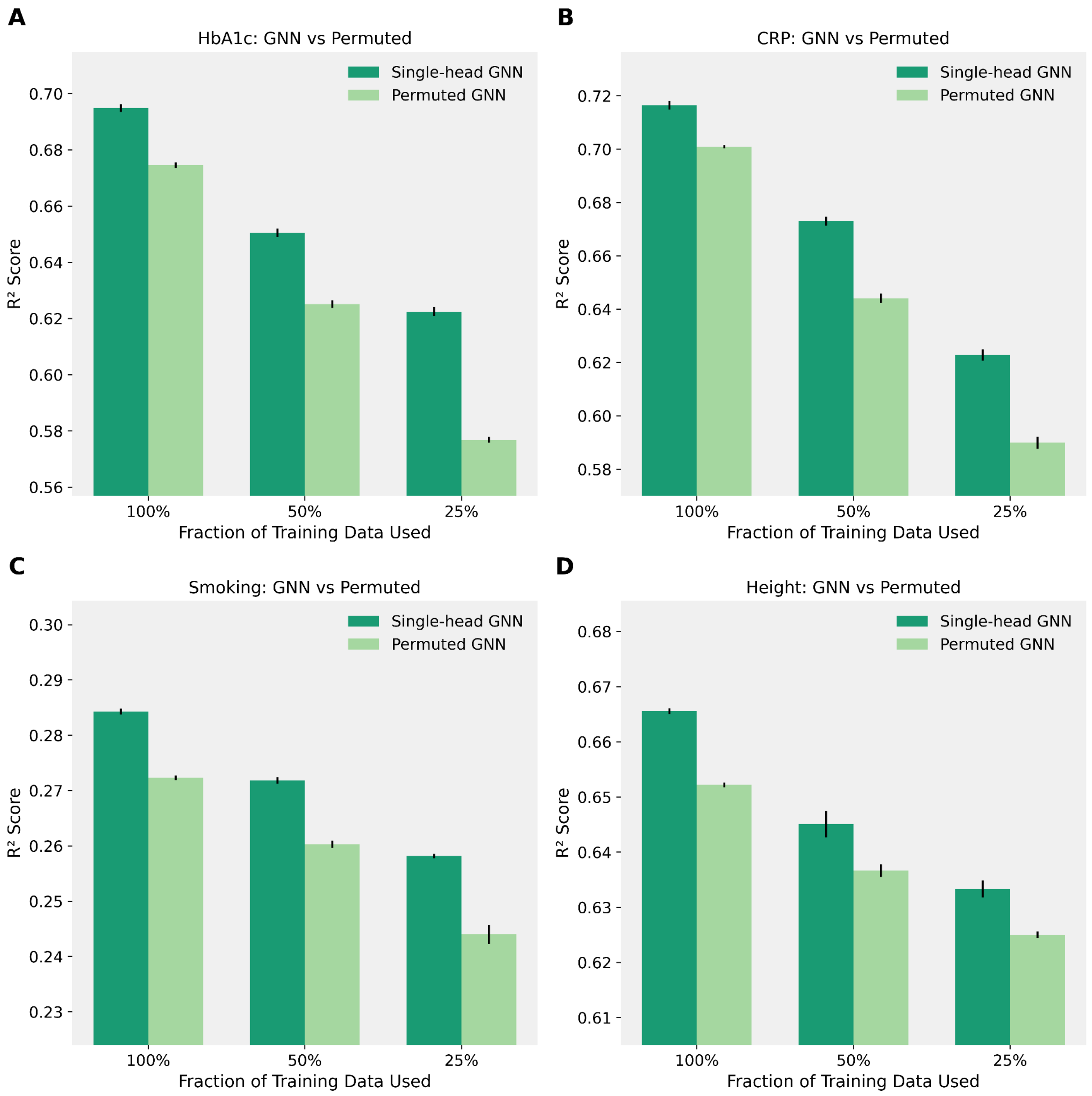

Supplementary Figure 1. Comparative evaluation of single-head graph neural network architectures and baseline models for multiple phenotype prediction.

The single-head GNNs, which operated on the protein graph, were trained on the full training data (100%) and smaller subsets of it (50%, 25%). Each model was compared to a corresponding permuted-graph baseline (see "Baseline models" section in Methods) in which protein names were permuted to remove biological meaning from the graph. Bars represent mean test R^2 across 10 independent training runs (± S.E.M.). The phenotypes evaluated were **(A)** glycated hemoglobin (HbA1c; UKBB Field 30750), **(B)** blood C-reactive protein (CRP) measured from serum biochemistry (UKBB Field 30710), **(C)** pack years smoked (UKBB Field 20161), and **(D)** standing height (UKBB Field 50). When training with the full training set, test R^2

medRxiv preprint doi: https://doi.org/10.1101/2025.11.23.25340814; this version posted November 25, 2025. The copyright holder for this preprint (which was not certified by peer review) is the author/funder, who has granted medRxiv a license to display the preprint in perpetuity.

It is made available under a CC-BY 4.0 International license .

values obtained from ridge regression (not shown) for HbA1c, blood CRP, pack years smoked, and height were 0.6231, 0.6687, 0.2741, and 0.6721, respectively.

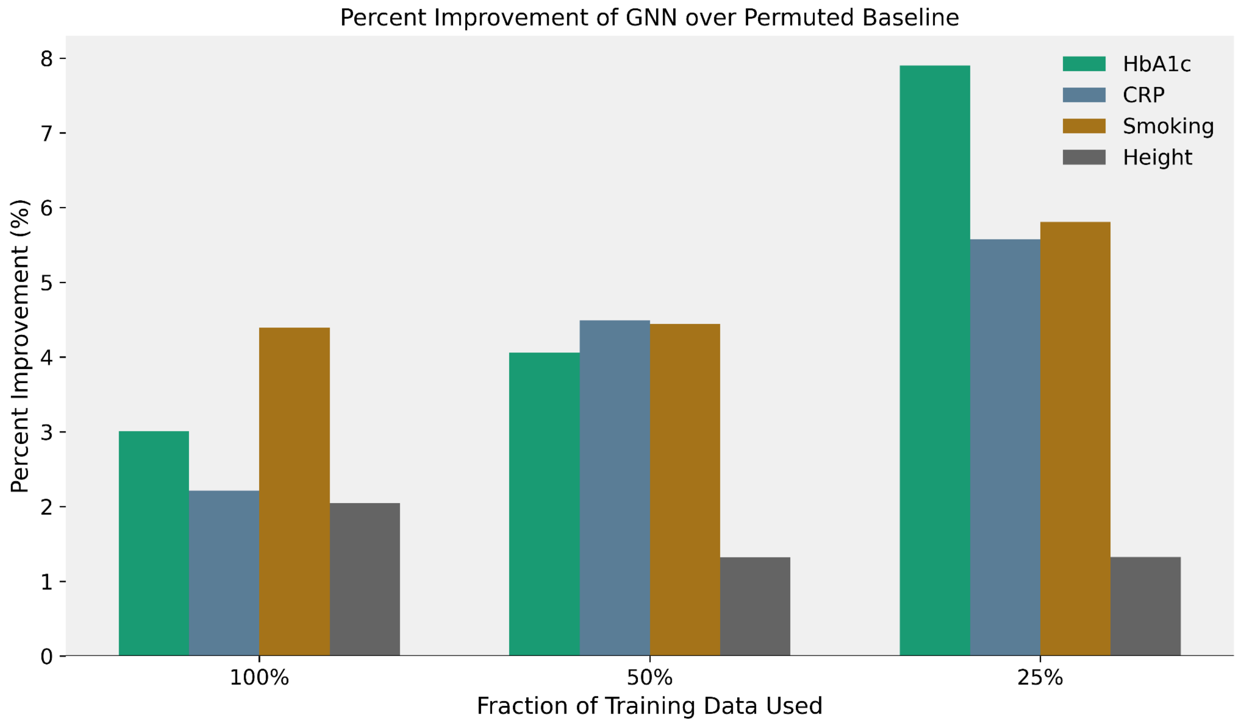

Supplementary Figure 2. Performance of single-head GNNs compared to permuted GNNs across multiple phenotypes and training data fractions.

The single-head GNNs, which operated on the protein graph, were trained on the full training data (100%) and smaller subsets of it (50%, 25%). Each model was compared to a corresponding permuted-graph baseline (see “Baseline models” section in Methods) in which protein names were permuted to remove biological meaning from the graph. Mean percent improvement in test R^2 (represented by bars) was computed for each model relative to its permuted-graph baseline. The phenotypes evaluated were **(A)** glycated hemoglobin (HbA1c; UKBB Field 30750), **(B)** blood C-reactive protein (CRP) measured from serum biochemistry (UKBB Field 30710), **(C)** pack years smoked (UKBB Field 20161), and **(D)** standing height (UKBB Field 50).

medRxiv preprint doi: https://doi.org/10.1101/2025.11.23.25340814; this version posted November 25, 2025. The copyright holder for this preprint (which was not certified by peer review) is the author/funder, who has granted medRxiv a license to display the preprint in perpetuity.

It is made available under a CC-BY 4.0 International license .

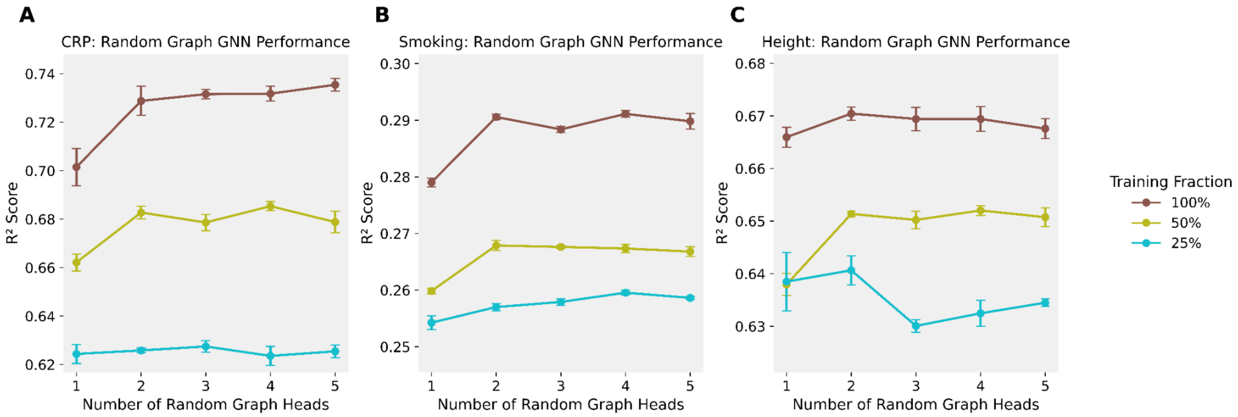

Supplementary Figure 3. Performance of GNNs trained on random graph structures across additional phenotypes not presented in the main paper.

GNNs were trained using randomly generated bipartite graphs containing between one and five random graph heads, with 100%, 50%, and 25% of the training data used for each configuration. Points represent mean test R^2 across 5 independent training runs (± S.E.M.). The phenotypes evaluated were **(A)** blood C-reactive protein (CRP) measured from serum biochemistry (UKBB Field 30710), **(B)** pack years smoked (UKBB Field 20161), and **(C)** standing height (UKBB Field 50).
